# Acupuncture Deqi Intensity and Propagated Sensation along Channels May, Respectively, Differ due to Different Body Positions of Subjects

**DOI:** 10.1155/2013/897048

**Published:** 2013-10-23

**Authors:** Xiang-Zhu Chen, Yun-Kuan Yang, Jie Yang, Ming-Xiao Yang, Shu-Wei Feng, Xiao-Juan Hu, Xiao Luo, Yue Feng, Fan-Rong Liang

**Affiliations:** Chengdu University of Traditional Chinese Medicine, 37 Shi-Er-Qiao Road, Jinniu District, Chengdu, Sichuan 610075, China

## Abstract

Acupuncture as an essential component of complementary and alternative medicine is gradually recognized and accepted by the mainstream of contemporary medicine. For obtaining preferable clinical effectiveness, Deqi is commonly regarded as efficacy predictor and parameter which is necessary to be achieved. Influential factors for acupuncture efficacy, like Deqi sensation as well as propagated sensation along channels (PSCs), enjoyed a long history in acupuncture basic research. Concerning this study, taking into account different positions on acupuncture Deqi sensation and PSCs, we would like to attest whether different body positions for subjects during needling procedure yield differed acupuncture Deqi sensation, particularly in terms of intensity, and PSCs. *Methods*. We used self-controlled method and selected 30 healthy subjects to perform needle insertion at Futu point (ST32) bilaterally. Then they were instructed to record the value of intensity of acupuncture sensation and the length and width of PSCs after removing the needle. *Results*. In regard to intensity of Deqi, kneeling seat position is stronger than supine position, accounting for 90% of the total number of subjects. In length of PSCs, kneeling seat position is greater than supine position, accounting for 56.7%. In width of PSCs, kneeling seat position is greater than supine position, accounting for 66.7%. *Conclusion*. Our findings show that needle inserting at Futu point (ST32) in kneeling seat position achieve better needle sensation and provide reference for clinical.

## 1. Introduction

Acupuncture is the most popular component of traditional Chinese medicine in western countries. The therapeutic effect of acupuncture appears when a needle sensation of Deqi is achieved and PSCs is arrived. Deqi was believed to be an indispensable composition of acupuncture treatment [1]. As we know, Deqi, a composite of unique needle sensation, namely, qi sensation under the tip of needles and qi sensation at affected region, is essential for clinical efficacy according to traditional Chinese medicine. It is described as a sensory perception of varying character and is mostly ascribed to metal needle acupuncture. The importance of Deqi has been investigated on the modern biological and medical framework in recent years [2–14]. Such work was started a couple of years ago by Kou et al. [15]. This research group asked 11 subjects to use the Visual Analog Scales (VASs) to quantify the Deqi sensations experienced during acupuncture administration. A principal component analysis identified 5 components: numbness, pressure, warmth, heaviness, and radiating paraesthesia. Four points (ST36, SP10, LI11, and GV14) were marked to acupuncture, involving needle insertion, stimulation, and removal, and finally completed the VASs. The results indicated that acupuncture significantly induced higher VAS values for numbness, pressure, warmth, and radiating paraesthesia, but not heaviness than the placebo across three treatment sessions. In summary, the induction of Deqi in each treatment session is an important factor for the physiological outcomes of repeated acupuncture treatment, and 10-fold VASs (VAS; with 0 being no sensation and 10-fold the strongest sensible sensation) offer an objective way to assess different levels of sensation between subjects. There have been some other attempts to investigate the quality of Deqi sensations. Kong et al. [4] introduced the “Subjective Acupuncture Sensation Scale” (SASS), which includes nine descriptors of Deqi sensation based on traditional and contemporary literature and one supplementary row for subjects to describe perceptions in their own words. Using this instrument, they were able to show significant correlations between the feeling of numbness as well as soreness and analgesic effect of acupuncture. Later on, a revised version of the SASS and the MASS (MGH Acupuncture Sensation Scale) was developed in cooperation with other research groups, which includes two supplementary scales for spreading sensation and anxiety [1]. Compared with SASS and MASS, VAS is more sensitive and suitable in this experiment. SASS and MASS give only a rough estimate of the spreading/radiation experienced by the single subject. To gain a deeper understanding of Deqi, it is crucial to measure its exact course and compare it between subjects. 

 PSCs, namely, arrival of qi at diseased areas, are the most common phenomenon of meridians and collaterals that feelings of soreness, numbness, distending, heaviness, and other special feelings appear, starting from the stimulation point inserted by manual, electrical pulses, and so on, conducting along the meridian recorded in the textbook. The phenomenon of PSCs has been extensively investigated by Chinese biologists and medical workers in recent years [16]. Some studies provide an objective basis for the phenomena of channels and suggest that the phenomenon of PSCs is an objective fact in human body [17]. This paper of Liu et al. reported the observed 23 subjects about changes of the infrared thermal image of upper extremity induced by needle “Hegu” (LI4), “Daling” (P7), and “Neiguan” (P6) points. The results indicated that 10 out of 23 cases whose higher and middle temperature bands of upper extremity distributed along the “Dachang” (Large Intestine) channels, when they felt phenomena of propagated sensation along the “Dachang” channels induced by needle “Hegu” points. Those effects did not appear in the subjects who did not feel PSC along the “Dachang” channels induced by needled points. So the phenomenon of PSCs is an objective fact in human body. 

Deqi intensity and PSCs are influential factors for acupuncture efficacy, and acupuncture point with different body position may lead to different Deqi intensity and PSCs. We aim to certify whether different body positions for subjects during needling procedure may produce differed acupuncture Deqi sensation, especially in terms of intensity, and PSCs.

## 2. Methods

### 2.1. Study Acupuncture Point

“Futu (ST32)” is first seen in the Meridian chapter of the Miraculous Pivot (*Lingshu*). ST32 is located in strands of rectus muscle of thigh before uplift, which belongs to the Stomach Meridian of Foot-Yang Ming in the textbook “A-B Classic of Acupuncture and Moxibustion” [18]. The primary tissue under the skin areas of ST32 point is, in order, superficial fascia, deep fascia, and rectus femoris, accessing into femoralis. The skin areas are dominated by nerves including femoral nerve anterior cutaneous branches and lateral femoral cutaneous nerve. In the deep intramuscular, there are muscle branch of the femoral nerve and the descending branch of lateral femoral circumflex artery and vein delivered by vein. The main effect of ST32 point is removing wind dampness, activating the channels and collaterals, and alleviating pains. ST32 has a widely clinical application: like cold waist and knee pain, lower limb paralysis, women all disease, hernia, abdominal pain, addiction rash, beriberi, knee arthritis, measles, bubo, and so forth [19–22]. While ST32 can be inserted in two different positions, and each of them might acquire good needle sensation and PSCs. Due to the widely clinical treatment and two different acupuncture needle positions of ST32 point which is according with our studies, so we choose ST32 point as our object of the experiment. But which position might gain the better clinical effect are studied and compared in this paper. 

### 2.2. Study Subjects

We selected 30 subjects to perform needle insertion at ST32 point bilaterally. The inclusion criteria are (1) healthy, (2) aged 18~25, (3) no limitation on gender, (4) normal-weight (BMI 18.5~24.0) and (5) flexibility in lower limbs and no trauma in the skin areas of ST32 point. The exclusion criteria are (1) suffered from cardiovascular disease, tristimania, and coronary heart disease, (2) the legs with trauma, and (3) feeling disorder. All of the subjects were recruited from Chengdu University of Traditional Chinese Medicine and undergraduate. They knew all the procedures of our experiment, and volunteer joined our experiment. We utilized self-controlled method in this study. The most commonly used method was adopted to judge the current criteria weight—calculated weight index (BMI). Weight index = weight (kg)/the square of height (⊣).

### 2.3. Acupuncture Stimulation and Procedure

Once the subjects were prepared well and reached a steady state, acupuncture stimulation was performed. ST32 point is located in strands of rectus muscle of thigh before uplift. For manual acupuncture stimulation, sterile single-use needle (length: 50 mm, diameter: 0.25 mm; Tian Xie, Suzhou, China) and fingernail-pressing were adopted to insert perpendicularly to the skin to a depth of approximately 40 mm at the Futu point (ST32) on the right side with supine position. Needle manipulation was practiced until a Deqi sensation was reported by the subject. The needles were twisted 1 min, then lifted and thrusted 1 min within a narrow range (1 mm) after acupuncture stimulation Deqi. The stimulation was performed immediately after inserting the needle, 10 min later, and before removing the needle, always by the same acupuncturist. After 20 min, the needles were removed. Then, all the subjects were requested to relax themselves and have a minor movement, especially the right legs. After 10 minutes later, when the subjects were asked no needle sensation and PSCs, we began the needle insertion on the left side with kneeling seat position. All the acupuncture manipulate and process are the same as the right side. And All the acupuncture manipulate are the same acupuncturist. All points were identified by anatomical marks based on description in textbooks [23]. 

### 2.4. Visual Analog Scale (VAS)

Subjects were instructed to record the value of intensity of acupuncture sensation and the length and width of PSCs after removing the needle in two different positions. VAS is objective, an easy, and reliable way to assess the intensity of needle sensation of Deqi [15]. At the end of acupuncture stimulation, the subject was asked if each Deqi sensation (soreness, numbness, distending, heaviness, and radiating paraesthesia) listed in a questionnaire occurred during stimulation and if it is present to rate its intensity on a numerical scale of 1–10 [24]. 0 means no needle sensation, 5 said moderate needle sensation, and 10 said the strongest (intolerable) needle sensation. Subjects make a mark on corresponding parts of the straight line, and from 0 to the distance of the mark is the needle sensation rating scores according to the needle sensation degree. Instruct the subjects to describe the conduction properties and direction of needle sensation, with the data collector to measure the length and width of needle sensation according to their own feelings. The characteristics of the VAS are summarized in Table 1. VAS intensity on a numerical scale is divided into 1–10, while data analysis of the experimental results can be segmented into [0,3], (3,6], and (6,10] points.

### 2.5. Statistical Analysis

The data were analyzed by using several tables to compare Deqi sensation intensity and conduction properties of PSCs by acupuncture ST32 point. Percentage method was used to analyze data from the study.

Acmesthesia scale:


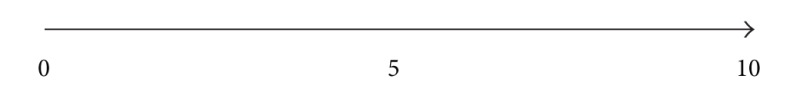


## 3. Results

Tables 1–4 demonstrate the total results from the parameters (mean values ± standard deviation) of acupuncture ST32 point in Deqi sensation intensity and PSCs.

### 3.1. Trial Characteristics

#### 3.1.1. Needle Sensation Intensity

The key outcomes from each individual trial are summarized in Table 2. All trial found that 30 subjects had appeared different degrees of needle sensation intensity after acupuncture. In (3,6] points (namely, greater than 3 point and less than or equal to 6 point), 18 subjects in supine position accounted for 60.0% of the total subjects; there were 7 subjects who accounted for 23.3% of the total subjects in (6,10] points. Kneeling seat position in (3,6] and (6,10] points had a large proportion; both, respectively, accounted for 46.7% of the total subjects. Supine position was mostly in the moderate intensity, while kneeling seat position was mostly in the medium and high intensity from the analyzed data and the standard of VAS scores. Thus, kneeling seat position might be stronger than supine position in needle sensation intensity. 

#### 3.1.2. The Length of PSCs

The key outcomes from individual trial are summarized in Table 3. All trials showed that 30 subjects had different degree of length of PSCs after acupuncture. 26 subjects in supine position accounted for 86.7% of the total subjects in [0, 12] cm; there were 4 subjects who accounted for 13.3% of the total subjects in (24,36] cm. There were 28 subjects of kneeling seat position and accounted for 93.4% of the total subjects in [0, 12] cm. Kneeling seat position in (12,24] and (24,36] had only 1 subject for each; both, respectively, accounted for 3.3% of the total subjects. Length of both supine and kneeling seat position were blow 12 cm, and supine position might be longer than kneeling seat position from the analyzed data but is not obvious.

#### 3.1.3. The Width of PSCs

The key outcomes from individual trial are summarized in Table 4. All trials showed that 30 subjects had different degree of width of PSCs after acupuncture. There were 15 subjects who accounted for 50.0% of the total subjects in [0, 2] cm, and the percentage was the largest; 14 subjects in supine position accounted 46.7% of the total subjects in (4,6] cm. There were 14 subjects of kneeling seat position who accounted for 46.7% of the total subjects in (2,4] cm, and percentage was the largest; 11 subjects in kneeling seat position accounted for 36.7% of the total subjects in [0, 2] cm; there were only 5 subjects who accounted for 16.6% of the total subjects in (4,6] cm. Width of both supine and kneeling seat position was blow 24 cm, but the percentage of kneeling seat in (4,6] was obviously greater than the supine position, so kneeling seat position might be greater than supine position in width of PSCs. 

### 3.2. Quality Assessment and Sensitivity Analysis

Overall, through the analyzed data in the experiment, kneeling seat position might be stronger than supine position in needle sensation intensity while needling ST32 point in two different positions. We know that width of kneeling seat position in PSCs might be greater than supine position; length of kneeling seat position might be greater than supine position within a scope of certain length, but more than a certain length range, kneeling seat position might be shorter than supine position. 

## 4. Discussion

This study compared the acupuncture Deqi intensity and PSCs between supine position and kneeling seat position, while needling the ST32 point. Our findings suggest that acupuncture needle at the ST32 point in different positions may produce different needle sensation intensity and PSCs. More specifically, acupuncture at the ST32 point in kneeling seat position may provide more effective needle sensation intensity and PSCs for clinical therapy.

Deqi, comprising mostly subjective needle sensations during acupuncture, is traditionally considered as a very important component for the possible therapeutic effect of acupuncture. Deqi sensation involving numbness, heaviness, aching, dull pain, and tingling are reported in most studies [24–29]. PSCs are the most common phenomenon of meridians and collaterals, and that feelings of soreness, numbness, distending, heaviness, and other special feelings appear. It is important for acupuncturist to apply the traditional and modern acupuncture methods to stimulate and promote the PSCs because of its arrival of qi at diseased areas. So as indispensable parts and measurements of therapy, Deqi sensation intensity has been measured by VAS scale. But till now, there is no consensus on the research methods to assess the credibility of PSCs. In order to gain a deeper understanding of PSC, it is crucial to measure its exact course and compare it between subjects. It is also important for acupuncturist to apply the traditional and modern acupuncture methods to stimulate and promote the Deqi sensation and PSCs which are significant to improve the clinical effect. 

 With the increasing access to modern clinical application research, it is particularly effective in treatment of acute stomach pain by acupressure at the ST32 point in medical practice. During treatment, ST32 in right side is more sensitive than left side, and the curative effect is quick [19]. If the disease is urgent, ST32 can be selected on both sides. In addition, some clinical research reported about treating disease by needling ST32, and the research results showed that 72 cases of shoulder coagulation disorder could be treated by needling ST32, and totally effective rate was 90% [19, 22]. In terms of acupuncture needle-retaining technique (including 20 min, 40 min, and 60 min groups), choosing ST32, Zusanli, and other points to use acupuncture needle, the effective rate of 60 min group was reached 85.37% while doing acupuncture therapy for the 251 cases of stroke patients [30]. 

Acupuncture needle at ST32 point mainly produced needle sensation of Deqi (feeling of soreness, numbness, distending, and heaviness) and stimulates PSCs due to the location of ST32 in clinical. There are two different positions of needling ST32 point, while the needle sensation intensity and PSCs are different produced by different positions, so the clinical effect might be different too. We can choose the effective position of point in which needle sensation intensity and PSCs are obvious in order to improve the clinical effect. That is why we did this experiment. We can know that kneeling seat position might be stronger than supine position in terms of needle sensation intensity and PSCs in our studies for three reasons. This is proved from our experimental data which is objective existence. The other reason might be acupuncture cumulative effect. Needle sensation is mainly formed in the deep tissue, and acupuncture stimulation may have an effect on the nerve bundle and deep variety of receptors (mainly for free sensory endings and muscle spindle) as well as the descendant of neural devices on the walls of blood vessels; all of them are through nervus cerebrospinalis to central nervous system. So when we first needled ST32 point of the right side in supine position, the central nervous system produced sensation of Deqi, after 10 minutes later, we needled the left side in kneeling seat position and the sensation of Deqi was also afferent to the central nervous system. The second acupuncture sensation might be stronger because the second Deqi sensation was different to the central nervous system, while the first Deqi sensation was not fading away completely in central nervous system. The third reason is that when we needle ST32 point in supine position and kneeling seat position, it differs: thigh muscle of kneeling seat position is in the pull tight state, the feeling of operate finger is also relatively heavy, and the muscle in areas of point exits pull stimulation, so Deqi sensation of kneeling seat position might be stronger than the supine position in which muscle is in relaxed state. So from above three reasons (including subjective sensation and objective fact), we can choose kneeling seat position to needle ST32 point in clinic: on one hand it might improve the clinical acupuncture sensation and PSCs, and on the other hand it might treat the disease in effect.

We use VASs to measure the needle sensation in our studies. Up to date, VASs have been used to measure Deqi sensation in both male and female [4]. VASs are objective, an easy, and reliable way to assess the intensity of needle sensation of Deqi [15]. Overall, 30 subjects had different degree of needle sensation intensity after acupuncture. Comparing the analyzed data from Table 2, we know that kneeling seat position might be stronger than supine position no matter the needle intensity or the quantity of the subjects which felt the Deqi sensation. From Tables 3 and 4, 30 subjects had different degree of length and width of PSCs after acupuncture. The length and width of kneeling seat position might be stronger than supine position in PSCs according to the measurement of length and width by acupuncturist from the own description of subjects. 

We had several limitations in this experiment. One of the limitations is insufficient sample size. Another limitation is operation technique limitation. Different selection of acupuncture technique and operating correctly or not are vital to control and motivate PSCs in this study. The third limitation is local blood stagnation of limbs. It takes about 4~5 minutes to keep kneeling seat position leading to leg numbness which may affect the results. The fourth limitation is different needle sensation between left and right side. On either side of the human nervous system, blood vessel and muscle tissue in ordinary life may be not suffered the same damage and produced different tiny lesions between left and right side which may cause different needle sensation on both sides of human body. So further study should enlarge the sample size, standardize the acupuncture technique, improve the experimental method, and choose other point with different position in order to make the results more statistically significant and persuasive.

## 5. Conclusions

Through the analyzed data on the experiment, the following conclusions can be drawn from the results of this transcontinental experimental acupuncture study.

- While needling ST32 point in two different positions, the sensation intensity of kneeling seat position might be stronger than the supine position. Founded in length of PSCs, kneeling seat position might be greater than supine position, and in width of PSCs, kneeling seat position might be also greater than supine position.
- The study shows that acupuncture needle at Futu point (ST32) in kneeling seat position might achieve better needle sensation and provide reference for clinical.
- The above conclusion might exist acupuncture cumulative effect; namely the kneeling seat position might be greater than supine position.

## Figures and Tables

**Table 1:** Standard of VAS score.

0 point	Painless
3 points below	Patient with mild pain that can be endurable
4 points–6 points	Patient with pain and affecting sleep which are tolerable
7 points–10 points	Patient with gradually intense pain which is painful

**Table 2:** Segmented data comparison about needle sensation intensity in two different positions.

Section (point)	Supine position		Kneeling seat position	
	Total number	Number (percentage)	Total number	Number (percentage)
[0,3]	30	5 (16.7%)	30	2 (6.6%)
[3,6]	30	18 (60.0%)	30	14 (46.7%)
[6,10]	30	7 (23.3%)	30	14 (46.7%)

**Table 3:** Segmented data comparison about length of PSCs in two different positions.

Section (cm)	Supine position		Kneeling seat position	
	Total number	Number (percentage)	Total number	Number (percentage)
[0, 40]	30	26 (86.7%)	30	28 (93.4%)
[40,80]	30	0 (0%)	30	1 (3.3%)
[80,120]	30	4 (13.3%)	30	1 (3.3%)

**Table 4:** Segmented data comparison about width of PSCs in two different positions.

Section (cm)	Supine position		Kneeling seat position	
	Total number	Number (percentage)	Total number	Number (percentage)
[0, 6.67]	30	15 (50.0%)	30	11 (36.7%)
[6.67,13.33]	30	14 (46.7%)	30	14 (46.7%)
[13.33, 20]	30	1 (3.3%)	30	5 (16.6%)
